# Special Issue: “Skin Disease and Comorbidities”

**DOI:** 10.3390/jcm10245754

**Published:** 2021-12-09

**Authors:** Keiichi Yamanaka

**Affiliations:** Department of Dermatology, Graduate School of Medicine, Mie University, 2-174 Edobashi, Tsu, Mie 514-8507, Japan; yamake@med.mie-u.ac.jp

The skin is one of the largest immune organs that involve innate and acquired immune systems, and is able to respond to internal and exogenous stimuli, producing a large amount of inflammatory cytokines, resulting in systemic inflammation. Several studies have shown that severe inflammatory skin disorders are connected to systemic complications, such as cerebro-arteriosclerosis, cardiomyopathy, abnormal fat metabolism, renal sclerosis, and systemic amyloidosis, leading to intimate relations between skin inflammation and complications, a concept of inflammatory skin march [1]. In psoriasis, one of the intractable inflammatory cytokine-mediated skin disorders, the average life span is 6 years shorter compared to that of the population without history of psoriasis mainly due to the cerebro-cardiovascular complications [2]. Furthermore, the eczema patients have increased risk for cardiovascular disorders [3]. Statistics have shown that atopic dermatitis and psoriasis patients have a high complication rate of coronary artery disease and cerebrovascular disease and often to be fatal [4,5,6,7].

Research on mice is ongoing, and the mechanism has been elucidated. Due to the release of cytokines at the site of interaction between skin constitute cells and inflammatory WBCs, atherosclerosis may be induced [8]. Atherosclerosis was not only detected in the abdominal aorta, but also in the peripheral basilar arteries, and these abnormalities were ameliorated by the administration of antibody against inflammatory cytokine [9]. The adipose tissue is also influenced, leading to the burning of adipocytes and the release of adipocytokines, which contribute to the systemic inflammatory cascade [10]. The osteoporosis may be a complication due to a decrease in the vascular network of the bone and the number of osteoblasts, and an increase in osteoclasts [11]. Male infertility may be related to sperm hypoplasia caused by an increase in inflammatory cytokines from skin lesions [12]. However, these data need to be verified in humans.

In the current Special Issue, clinical evidence and cases focusing on systemic inflammatory changes and systemic organ diseases complicated by inflammatory skin disorders were reported and discussed. 

In the psoriasis field, Yamazaki et al. performed coronary computed tomography angiography (CCTA) in 88 patients with psoriasis and the ankle–brachial pressure index (ABI) for 44 of these patients. CCTA abnormalities were identified and compared to healthy controls. In the patients with abnormal results for both ABI and CCTA, the rates of CCTA vascular lesions were significantly higher, also revealing a correlation between CCTA and ABI in psoriasis patients [13].

A multifunctional protein, osteopontin (OPN) may contribute to the development of atherosclerosis and metabolic syndrome (MetS). Bartosinska et al. assessed the correlation between OPN concentration in the peripheral blood and the presence of MetS as well as its particular components in the psoriasis patients. Psoriasis patients with MetS had significantly higher obesity, systolic blood pressure, TG, CHOL/HDL, LDL/HDL, and TG/HDL ratios than psoriasis patients without MetS. OPN serum concentration was significantly higher in psoriasis patients than in the heartly controls [14].

As one of the facial inflammatory dermatosis, rosacea has been linked to manifest ocular surface changes, such as blepharitis and conjunctivitis. A multi-institutional case-control study revealed a notable association between rosacea and blepharitis, conjunctivitis, glaucoma, dry eye syndrome, and chalazion in the Korean patient population [15].

Systemic sclerosis (SSc) is a connective tissue disease characterized by multisystem fibrotic vasculopathic disorder with autoimmune abnormalities. Calponin 3 plays a role in the cell motility and contractibility of fibroblasts during wound healing in the skin. The serum calponin 3 level was significantly higher in SSc patients than in healthy controls. The modified Rodnan total skin thickness score was significantly higher in the elevated serum calponin 3 level group than in the normal level group. Elevated serum calponin 3 level was associated with skin sclerosis and arthralgia in SSc patients. Serum calponin 3 levels might act as a biomarker that reflects the severity of skin sclerosis and joint involvement in SSc [16]. On the other hand, serum thymus and activation-regulated chemokine (TARC) levels were also significantly elevated in patients with SSc, especially in those with the diffuse subtype compared with healthy controls. In particular, diffuse cutaneous type SSc patients with SSc-associated interstitial lung disease (ILD, SSc-ILD) showed higher TARC levels than those without SSc-ILD [17].

The temporal relationships between inflammatory bowel disease (IBD)-associated cutaneous manifestations and IBD have been uncertain. Hung et al. determined the association and temporal relationship between cutaneous manifestations and IBD. The risks of cutaneous manifestation before and after the diagnosis of IBD include atopic dermatitis, erythema nodosum, and aphthous stomatitis. IBD was also associated with the subsequent development of pyoderma gangrenosum, erythema nodosum, polyarteritis nodosa, and hidradenitis suppurativa [18]. 

Until now, dermatitis has been regarded as a simple reaction occurring in an isolated organ, the skin. However, as in the recent published reports and the papers listed in the current Special Issue, considering the possibility that cytokines produced from inflammatory skin sites may induce systemic inflammation, or that skin inflammation is one phenotype of systemic inflammation and further induces and amplifies systemic inflammation through activation of leukocytes and cells of internal organs, it may be necessary to consider aggressive treatment of dermatitis.
